# Detection of Multiple Intracellular Bacterial Pathogens in *Haemaphysalis flava* Ticks Collected from Hedgehogs in Central China

**DOI:** 10.3390/pathogens10020115

**Published:** 2021-01-23

**Authors:** Li-Zhu Fang, Si-Cong Lei, Zhi-Jian Yan, Xiao Xiao, Jian-Wei Liu, Xiao-Qing Gong, Hao Yu, Xue-Jie Yu

**Affiliations:** 1 State Key Laboratory of Virology, School of Health Sciences, Wuhan University, Wuhan 430071, China; 2018103050006@whu.edu.cn (L.-Z.F.); 2012302170018@whu.edu.cn (S.-C.L.); xiaoalltheway@gmail.com (X.X.); liujianwei@whu.edu.cn (J.-W.L.); 2017203050014@whu.edu.cn (X.-Q.G.); yu_h89@yahoo.com (H.Y.); 2 The First Affiliated Hospital, Sun Yat-sen University, Guangzhou 510000, China; 3 Sixth People’s Hospital, Qingdao 266000, China; qdlyyzj@163.com; 4 Lab Animal Research Center, Hubei University of Chinese Medicine, Wuhan 430000, China

**Keywords:** *Haemaphysalis flava*, hedgehogs, *Anaplasma bovis*, *Coxiella burnetti*, *Ehrlichia ewingii*, *Rickettsia raoultii*, *Rickettsia japonica* and China

## Abstract

Tickborne intracellular bacterial pathogens including *Anaplasma*, *Coxiella burnetti, Ehrlichia*, and *Rickettsia* cause emerging infectious diseases worldwide. PCR was used to amplify the genes of these pathogens in *Haemaphysalis flava* ticks collected from hedgehogs in Central China. Among 125 samples including 20 egg batches, 24 engorged females, and 81 molted male and female adult ticks, the DNA sequences and phylogenetic analysis showed that the minimum infection rate of the ticks was 4% (5/125) for *A. bovis*, 3.2% (4/125) for *C. burnetti*, 9.6%, (12/125) for *E. ewingii*, and 5.6% for *Rickettsia* including *R.* *japonica* (3.2%, 4/125) and *R. raoultii* (2.4%, 3/125), respectively. The prevalence of these pathogens was significantly higher in dead engorged females (83.3%, 20/24) than in eggs (5%, 1/20) and molted ticks (8.6%, 7/81). Our study indicated that *H. flava* ticks could be infected with multiple species of tickborne pathogens including *Anaplasma*, *C. burnetti*, *Ehrlichia*, and *Rickettsia* in Central China, and the prevalence of these pathogens was reduced during transovarial and transstadial transmission in ticks, suggesting that ticks may not be real reservoirs but only vectors for these tickborne pathogens.

## 1. Introduction 

Tickborne intracellular bacteria including *Anaplasma* spp., *Ehrlichia* spp., *Coxiella burnetti,* and *Rickettsia* spp. cause severe human diseases [1,2,3,4]. *Ixodidae*, the hard body ticks, play an important role in the maintenance and transmission of these tickborne pathogens [5]. Approximately 100 *Ixodidae* species have been identified in China. Because of the vast territory, complex geography, and different climates of China, tickborne diseases are prevalent in most parts of China and pose a serious public health threat [6,7,8].

*Haemaphysalis flava* ticks are widely distributed throughout Asia including in China, Japan, South Korea, and Vietnam [9,10,11,12]. Hosts of *H. flava* include domesticated animals such as horses (*Equus caballus*), pigs (*Sus scrofa domesticus*), dogs (*Canis lupus familiaris*), sheep (*Ovis aries*), cattle (*Bos taurus*), and wild animals such as hedgehogs (*Erinaceinae*), pandas (*Ailuropoda melanoleuca*), Siberian chipmunks (*Eutamias sibiricus*), Raccoon dogs (*Nyctereutes procyonoides*), water deer (*Hydropotes inermis*), and eastern roe deer (*Capreolus pygargus*) [13,14,15,16]. Previous studies had demonstrated that *H. flava* were positive to bacterial pathogens such as *Anaplasma bovis, Borrelia* spp., *Batonella*, *Francisella tularensis*, *Rickettsia japonica,* parasites such as *Babesia* spp. and *Toxoplasma gondii*, and viruses such as severe fever with thrombocytopenia virus and tickborne encephalitis virus [17,18,19,20,21,22,23,24,25]. These studies about *H. flava* tickborne pathogens were mainly carried out in Japan and South Korea. In China, only two studies in the Jiangxi and Hubei Provinces demonstrated that *H. flava* were positive to *R slovaca, R. japonica,* and unclassified *Ehrlichia* species [26,27].

The *Haemaphysalis flava* tick is widely distributed, and it is important to know the pathogens carried by *H. flava* in China. In this study, we investigated the prevalence of intracellular bacterial pathogens in *H. flava* ticks collected from hedgehogs in Hubei Province in Central China. 

## 2. Materials and Methods

### 2.1. Tick Samples

Ticks were pulled parallel to the skin surface by using fine-tipped tweezers from hedgehogs (*Erinaceus amurensis*) collected in October 2018 [28]. Hedgehogs were captured from forests near the cesspools in Xianning City, Hubei Province, China [29]. Xianning is located at 29°87′ north latitude and 114°28′ east longitude in Central China. The temperature ranges from −7 °C to 40 °C, with an annual average of 16.8 °C. All ticks were morphologically identified as *H. flava* [15], and nine ticks were randomly selected to amplify the 16S rRNA gene (*rrs*) with PCR for species confirmation as previously described [30]. Engorged ticks were kept in an incubator with 85% relative humidity at 25 °C for oviposition or molting [31].

### 2.2. PCR Amplification of Tickborne Pathogens in Ticks

For the detection of tickborne bacteria, the egg batches from each female were processed together, the dead engorged females were processed individually, and molted ticks (females and males) were processed in groups of six or seven ticks. Ticks were washed with distilled water and dried before DNA was extracted with the AllPrep DNA Mini Kit (Qiagen, Hilden, Germany) following the manufacturer’s instructions. Tick DNA was dissolved in 100 μL of DNase free water and stored at −80 °C.

Tick DNA was used for the PCR amplification of tickborne pathogens. The primers listed in Table 1 were used to amplify the *Anaplasma* 16S rRNA gene *(rrs*) and heat shock protein GroEL (*groEL*) genes, the *C. burnetti* outer membrane protein (*omp*) gene and isocitrate dehydrogenase (*icd*) gene, the *Ehrlichia rrs* and GltA (*gltA*) genes, and the *Rickettsia* 17-kDa protein gene, outer membrane protein A (*OmpA*) gene, *gltA* and *rrs*. The PCR cycles of outer and inner primers for each gene were one cycle of 5 min at 95 °C, followed by 35 cycles of 1 min at 95 °C, 1 min at 55 °C, and 1 min at 72 °C, and a final extension step of 10 min at 72 °C. Nuclease-free water was used as negative controls for each experiment.

PCR products were analyzed using 1.2% agarose gel electrophoresis and were detected with ethidium bromide staining under UV light. PCR products with the expected size were excised from the gels and extracted with a Gel Extraction Kit (Omega, Norcross, Georgia). The purified PCR products were cloned into PMD 19-T vectors (TaKaRa, Shiga, Japan), and the recombinant plasmids were sequenced bidirectionally. 

### 2.3. Phylogenetic Analysis

The sequence chromatograms and analysis were examined with Chromas and BLAST programs (http://blast.ncbi.nlm.nih.gov/Blast.cgi), respectively. Sequences were aligned and trimmed with MEGA7 (Philadelphia, PA, USA), and phylogenetic trees were constructed with MEGA7 using the maximum-likelihood method, with nucleotide sequences and bootstrap values that were calculated with 1000 replicates [38,41]. Only bootstrap values >50% were shown.

### 2.4. Statistical Analysis

All the statistical analyses was performed by Fisher’s exact test with SPSS (version 17.0) (Armonk, NY, USA), and *p* < 0.05 was considered to be a statistically significant difference.

## 3. Results

### 3.1. Tick Species 

A total of 125 ticks was collected from 15 hedgehogs, out of which 44 ticks were engorged adult females and 81 ticks were engorged nymphs. Of the engorged females, 20 females had oviposited and 24 females died before oviposition. All 81 engorged nymphs had molted into adult ticks regardless of sex. Ticks were morphologically identified as *H. flava* and confirmed with PCR amplification and DNA sequencing of the *rrs* gene. 

### 3.2. Phylogenic Analysis of Different Tickborne Intracellular Bacteria

**Figure 1:**
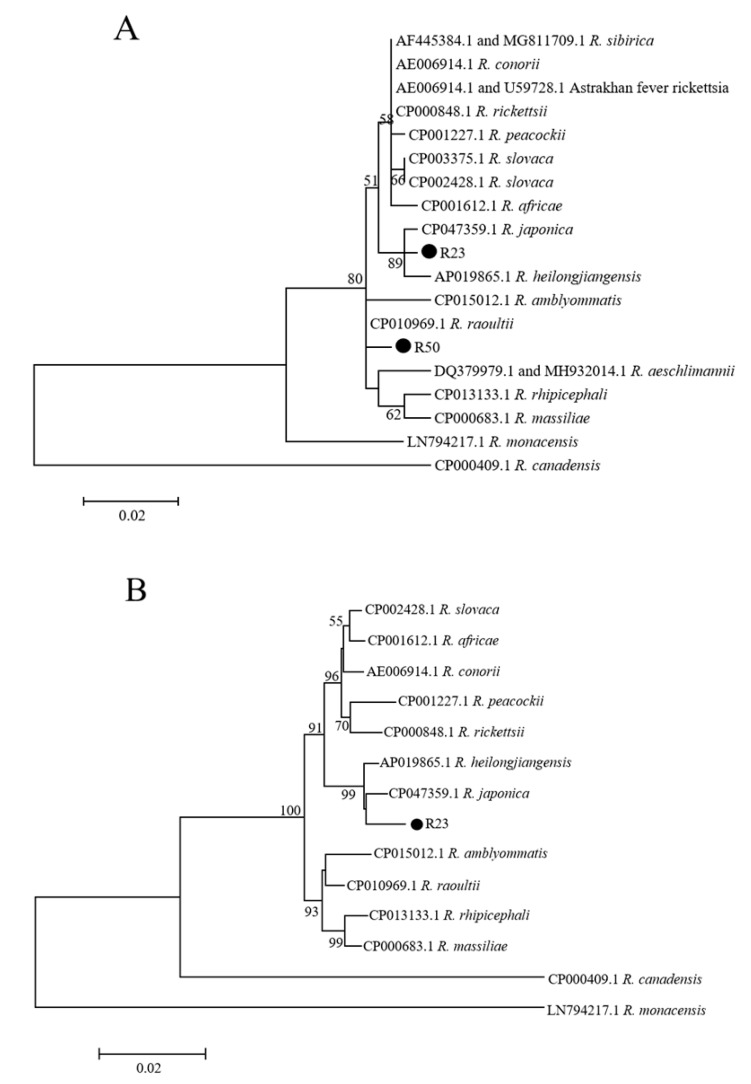
Phylogenetic tree of the *Rickettsia* species. The phylogenetic trees were constructed using (**A**) the concatenated sequences of the 17-kDa protein gene and *gltA* and (**B**) the concatenated sequences of *rrs*,17-kDa protein gene, *gltA*, and *ompA*. The tree was generated using the Maximum Likelihood method, the Kimura 2-parameter model, and 1000 replicates for bootstrap testing in MEGA 7.0 software. Only bootstrap values > 50% were shown. *Rickettsia* sequences obtained in this study are shown with dots. The scale bar indicates nucleotide substitutions per site. The *Rickettsia* species’ name and complete genome GenBank accession numbers of reference sequences are shown in each line.

*Rickettsia*: *Rickettsia* sequences were obtained from seven ticks and tick pools by PCR with primers of the 17-kDa protein gene. The DNA sequence analysis indicated that the 17-kDa protein gene positive samples were divided into two groups. Group 1 consisted of four sequences, which had the highest homology with *R. japonica* (GenBank: CP032049) (99.3–99.5% homologous), and group 2 consisted of three sequences, which had the highest homology with *R. raoultii* (GenBank: MH932036) (98.8–99.5%). Further amplification of the 17-kDa gene positive ticks and tick pools with primers of *ompA*, *gltA*, and *rrs* showed that ticks in group 1 had one, two, and three samples that were positive, respectively; and one tick pool in group 2 was positive with *gltA* primers. The rickettsial sequences in group 1 obtained with primers of *ompA*, *gltA*, and *rrs* had the highest homology with *R. japonica* with a 99.7%, 99.7–100%, 96.1%, and 99.4% homology to *R. japonica*, respectively, and the sequence in group 2 obtained with *gltA* primers had the highest homology with *R. raoultii* (99.1%). The phylogenetic analysis was performed with only one tick sample for each group, with the R23 tick representing group 1 and R50 tick representing group 2. A phylogenic analysis with concatenated sequences of the 17-kDa protein gene and *gltA* indicated that the R23 formed a cluster with *R. japonica* and *R. heilongjiangensis*; and R50 was in the same cluster as *R. raoultii* (Figure 1A). Due to the difficulty in differentiating between *R. japonica* and *R. heilongjiangensis*, they were further analyzed by using more sequences including all four genes we obtained in tick R23. A phylogenetic analysis with concatenated sequences of *rrs*, 17-kDa protein gene, *gltA*, and *ompA* showed that R23 was in the same cluster with *R. japonica* and *R. heilongjiangensis*, but closer to *R. japonica* (Figure 1B). 

**Figure 2:**
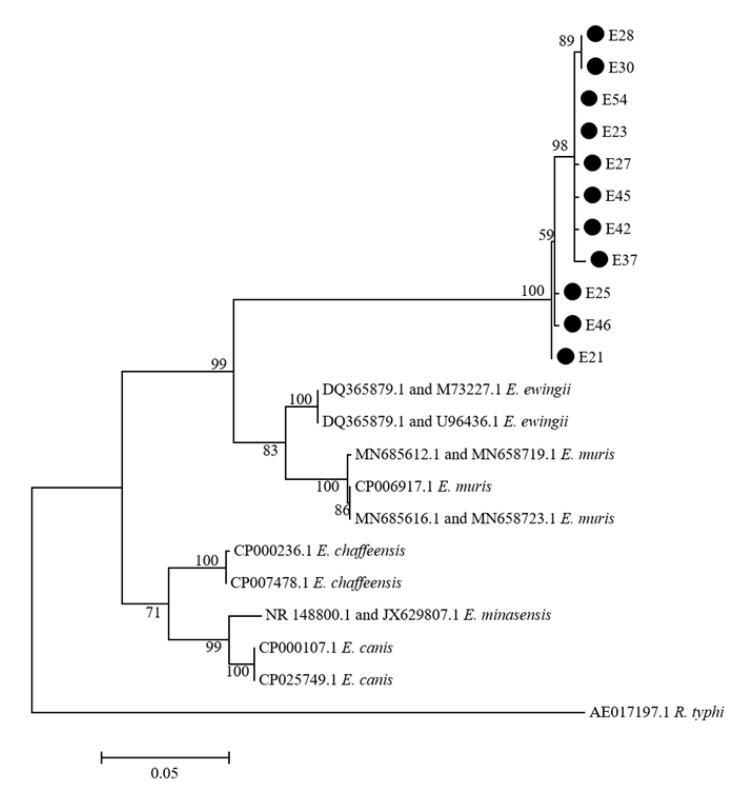
Phylogenetic tree of *Ehrlichia species*. The phylogenetic tree was constructed using the concatenated sequences of *rrs* and *gltA*. The tree was generated using the Maximum Likelihood method, the Kimura 2-parameter model, and 1000 replicates for bootstrap testing in MEGA 7.0 software. Only bootstrap values >50% were shown. *Ehrlichia* sequences obtained in this study are shown with dots. The scale bar indicates nucleotide substitutions per site. The *Ehrlichia* species’ name and GenBank accession numbers of reference sequences are shown in each line. For the *Ehrlichia* species without complete genome sequences, the GenBank accession numbers in the order of *rrs* and *gltA* were DQ365879.1 and M73227.1 for *E. ewingii*; DQ365879.1 and U96436.1 for *E. ewingii*; MN685612.1 and MN658719.1 for *E. muris*; MN685612.1 and MN658719.1 for *E. muris*; and NR 148800.1 and JX629807.1 for *E. minasensis*.

*Ehrlichia*: *Ehrlichia* sequences were obtained from nine individual ticks and three tick pools by PCR with *rrs* primers. The *rrs* sequences had the highest homology with *E. ewingii* (GenBank: U96436) (98.8–99.8%). The *rrs* positive ticks and tick pools were further amplified with *gltA* primers. The sequences obtained with *gltA* primers also had the highest homology with *E. ewingii* (GenBank: DQ365879) (90.3–90.9%). A phylogenic analysis with concatenated sequences of *rrs* and *gltA* indicated that all 11 *Ehrlichia* species were in the same cluster as *E. ewingii*, but in a distinct group, suggesting that this *Ehrlichia* species was a novel *Ehrlichia* species (Figure 2). 

**Figure 3:**
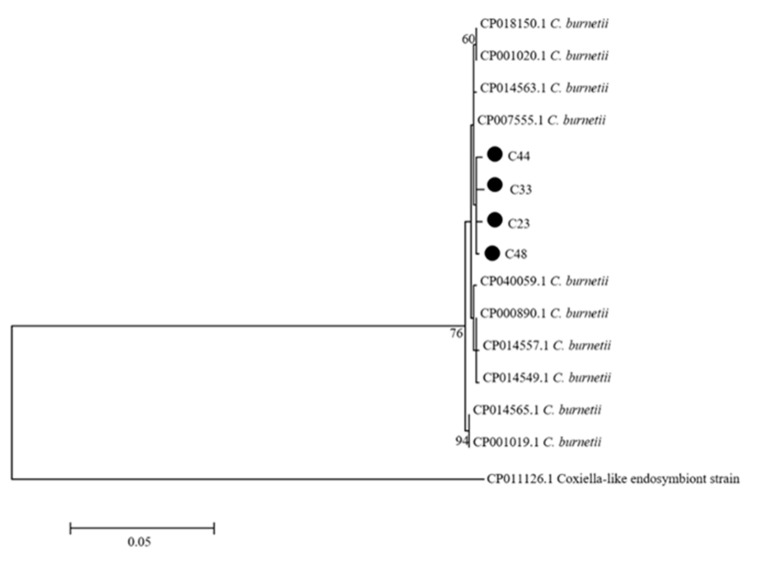
Phylogenetic tree of the *Coxiella* species. The phylogenetic tree was constructed based on the concatenated sequences of *omp* and *icd*. The tree was generated using the Maximum Likelihood method, the Kimura 2-parameter model, and 1000 replicates for bootstrap testing in MEGA 7.0 software. Only bootstrap values >50% were shown. *Coxiella* sequences obtained in this study are shown with dots. The scale bar indicates nucleotide substitutions per site. The *Coxiella* species’ name and complete genome GenBank accession numbers of reference sequences are shown in each line.

*Coxiella*: *Coxiella* sequences were obtained from three individual ticks and one tick pool by PCR with *omp* primers. The *omp* sequences from ticks had the highest homology with *C. burnetti* (GenBank: CP014563) (99.5–99.8%). The *omp* positive tick samples were further amplified with *icd* primers, which showed that all four tick samples were positive. The *icd* sequences from ticks were 99.8–100% homologous with *C. burnetii* (GenBank: CP040059). A phylogenetic analysis with concatenated sequences of *omp* and *icd* indicated that the four sequences from ticks were in the same cluster with *C. burnetti* (Figure 3).

**Figure 4:**
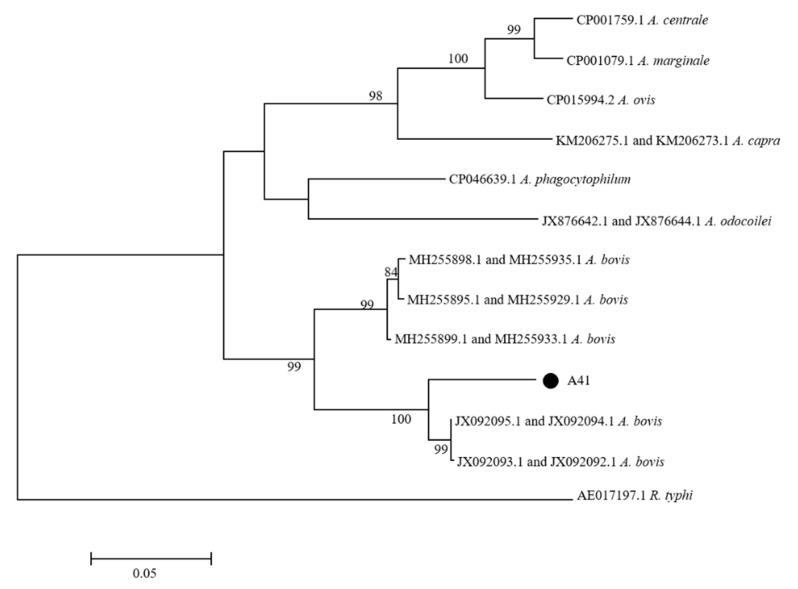
Phylogenetic tree of the *Anaplasma* species. The phylogenetic tree was constructed based on the concatenated sequences of *rrs* and *groEL*. The tree was generated using the Maximum Likelihood method, the Kimura 2-parameter model, and 1000 replicates for bootstrap testing in MEGA 7.0 software. Only bootstrap values >50% were shown. *Anaplasma* sequences obtained in this study are shown with dots. The scale bar indicates nucleotide substitutions per site. The *Anaplasma* species’ name and complete genome GenBank accession numbers of reference sequences are shown in each line.

*Anaplasma*: *Anaplasma* sequences were obtained from five ticks by PCR with *rrs* primers. Because the *rrs* sequences were short and had a poor specificity at first, the semi-nested primers were designed to prolong the *Anaplasma rrs* sequences of the positive ticks (Table 1). The *rrs* sequences were prolonged in four of five *rrs* positive ticks. The *rrs* sequences from ticks had the highest homology with *E. bovis* (GenBank: U03775) (96.3–100%). The *rrs* positive ticks were further amplified with *groEL* primers, which showed that only one tick sample was positive. The *groEL* sequence obtained from a tick was 84.6% homologous with *A. bovis* (GenBank: MH255898). A phylogenetic analysis based on the concatenated sequence of *rrs* and *groEL* indicated that the concatenated sequence was in the same cluster as *A. bovis* (Figure 4). 

### 3.3. Infection Rate of Tickborne Intracellular Bacteria in Ticks

The minimum infection rate (MIR) of pooled ticks was calculated by assuming only one tick was infected in a positive group, and the maximum infection rate (MAR) was calculated by assuming that all ticks were positive in a positive group. The MIR of all tickborne intracellular bacteria in the tested ticks was 22.4% (28/125) (Table 2). The MIR for all tickborne bacteria was 5% (1/20) for tick egg batches, and 83.3% (20/24) for dead engorged females. For molted adult ticks (females and males), the MIR was 8.6% (7/81) and the MAR was 60.5% (49/81). For a comparison of differences according to intracellular bacterial species, the prevalence of the novel *Ehrlichia* (9.6%, 12/125) was significantly higher than *A. bovis* (4%, 5/125) (*p* < 0.01), *C. burnetti* (3.2%, 4/125) (*p* < 0.01), *R. japonica* (3.2%, 4/125) (*p* < 0.01), or *R. raoultii* (2.4%, 3/125) (*p* < 0.01), but there was no significant difference among the last four bacterial species. For a comparison of differences according to groups of developmental stage, the prevalence of tickborne intracellular bacteria in dead engorged ticks was significantly higher than in molted adult ticks (*p* < 0.01) or eggs (*p* < 0.01), but there was no significant difference between molted adult ticks and eggs (*p* = 1).

GenBank deposition: the sequences of tickborne pathogens obtained in this study were deposited in GenBank with accession numbers: *A. bovis rrs*: MW275984–MW275987 and MN148605, and *groEL*: MW226869; *E. ewingii rrs* MN148606–MN148617, and *gltA*: MW226861–MW226866; *C. burnetti icd*: MW226857– MW226860, and *omp*: MW226877–MW226880. *Rickettsia* 17-kDa protein gene: MW226870–MW226876, *gltA*: MW226867 and MW226868, *rrs*: MW275981–MW275983, and *ompA* MW265948.

## 4. Discussion 

We collected 125 ticks from 15 hedgehogs in Hubei Province, Central China, and all ticks were *H. flava*. We found three genera of *Rickettsiales*, including: *Rickettsia*, *Ehrlichia* and *Anaplasma*, and *Coxiella burnetti* in different developmental stages of *H. flava*, including eggs, adult ticks, and dead engorged females. We hypothesized that ticks in different stages should have a similar infection rate of intracellular bacteria as they were all collected from 15 hedgehogs. However, our results showed that the prevalence of intracellular bacteria was significantly higher in dead engorged ticks than in eggs and adult ticks molted from nymphs, indicating that there were far more pathogens in every tick immediately after bloodmeal than after the transition to the next stage of development. The transovarial and transstadial transmission reduction in the prevalence of ticks can be observed in all detected nonrelated pathogens. The engorged females were not accidently killed during harvesting from hedgehogs due to their large body size. Even if the ticks were accidently killed, this could not explain why the infection rate of the intracellular bacteria was significantly higher in the dead ticks than in the live ticks. The significant difference in the infection rates between the dead engorged females, and the tick eggs or molted adult ticks may be explained by two possibilities. One possibility is the detrimental effect of intracellular bacteria on ticks, i.e., intracellular bacteria might be detrimental to the engorged adult females, causing the death of engorged female adult ticks during oviposition; another possibility is the transstadial blockage of intracellular bacteria in ticks, i.e., ticks obtained intracellular bacteria during feeding on hedgehogs, and the intracellular bacteria were lost during oviposition or molting due to these bacteria failing to be effectively transmitted transovarially or due to a transstadial blockage occurring in the molting ticks. A previous study showed that only 6% of *Ixodes ricinus* larvae could be infected by the European strain of tickborne encephalitis virus through transovarial transmission and that the Kyasanur forest disease virus could successfully pass transovarially in 59% of *Haemaphysalis spinigera* larvae [42]. Our previous study also demonstrated that 70% of *Haemaphysalis Iongicornis* ticks transovarially transmitted SFTSV and that only 20% transstadially transmitted SFTSV [30]. A previous study showed that if infected ticks were maintained on infection-free hosts for several generations, their pathogens would permanently disappear after 2–3 generations [43]. Our study and previous study suggested that ticks are not real reservoirs but only vectors for these tickborne pathogens.

An *Ehrlichia species* identified in this study was most closely related to *E. ewingii*, but in a distinct phylogenetic group, suggesting that it was a novel *Ehrlichia* species that needed to be further investigated. *Ehrlichia ewingii* had been reported to infect dogs and cause canine fever, thrombocytopenia, anorexia, polyarthritis, and central nervous system abnormalities [44]. The susceptible animal for this new *Ehrlichia* species needed to be investigated. *Coxiella burnetii*, the causative agents of Q fever, are broadly distributed in the environment. Livestock were identified as main reservoirs, which may infect people through their contaminative urine, feces, milk, and birth products. Our previous study had demonstrated that 12.2% of hedgehogs in Hubei Province were PCR-positive to *C. burnetii* [29]. Our studies indicated that both hedgehogs and their surface parasite ticks could serve as the animal host and vector for *C. burnetii.* Many kinds of animals could be infected by *A. bovis*, which caused animal abortions and the reduction of milk production and body weight, and which frequently led to death [45]. A previous study indicated the *A. bovis* infection of monkeys, suggesting that *A. bovis* may infect humans [46]. *Rickettsia japonica*, which was widely distributed in China (including in Henan [40], Anhui [7], Zhejiang [47], Shandong [48], and Hubei [26]) and *R. raoultii*, which was mainly reported around the border of China (like the Northeastern [49], Northwestern [50,51], Southwestern [52], Inner Mongolia [53], and Central [40] areas) belonged to the spotted fever group rickettsiae and could cause human fever, vomiting, nausea, maculopapular rash, and occasionally eschars at the site of inoculation [54].

To our knowledge, this is the first report about *C. burnetti, E. ewingii,* and *R. raoultii* in *H. flava*. *R. japonica*, *R. heilongjiangensis*, *Candidatus R. principis*, *R. felis*, and *R. helvetica* have been reported in *H. flava* in China, South Korea, and Japan [55,56,57,58,59]. These studies indicate that *H. flava* could transmit multiple rickettsial pathogens in Asia. 

In conclusion, *H. flava* ticks collected from hedgehogs in Central China were infected with multiple intracellular bacterial pathogens, including *R. raoultii*, *R. japonica*, *E. ewingii*, *C. burnetti*, and *A. bovis*. The diseases caused by these pathogens need to be monitored in China. 

## Figures and Tables

**Table 1:** Primers for the amplification of sequences of *Coxiella burnetti, Anaplasma* spp., *Ehrlichia* spp., and *Rickettsia* spp. from ticks.

Organisms	Primary/Nested	Primers	Primer Sequences	Target Gene	Amplicon Size	Reference
*Coxiella burnetti*	Primary	Omp1	AGTAGAAGCATCCCAAGCATTG	*omp*	438 bp	[32]
		Omp2	TGCCTGCTAGCTGTAACGATTG			
	Nested	Omp3	GAAGCGCAACAAGAAGAACA			
		Omp4	TGGAAGTTATCACGCAGTTG			
	Primary	BicdF1	CGGAGTTAACCGGAGTATCCA	*icd*	651 bp	[33]
		BicdR1	CCGTGAATTTCATGATGTTACCTTT			
	Nested	BicdF2	AGTTAACCGGAGTATCCATC			This study
		BicdR2	CTAAACGGCTCGTGCCTTCT			
*Anaplasma*	Primary	EC9	TACCTTGTTACGACTT	*rrs*	477 bp	[34]
		EC12A	TGATCCTGGCTCAGAACGAACG			
	Nested	EM87F	GGTTCGCTATTAGTGGCAGA			
		EM584R	CAGTATTAAAAGCCGCTCCA			
	Primary	fD1	AGAGTTTGATCCTGGCTCAG	*rrs*	742–1426 bp	[35]
		Rp2	ACGGCTACCTTGTTACGACTT			
	Nested	EHR16SD	GGTACCY * ACAGAAGAAGTCC			[36]
		EHR16SR	TAGCACTCATCGTTTACAGC			
	Primary	agroELwf	TTTGCCAGTTTTGGAAGGCG	*groEL*	473 bp	This study
		agroELwr	TTTCAGCGGATCCATCACCC			
	Nested	agroELnf	TGAGGGTGAAGCATTGAGCA			
		agroELnr	AGAGTGTACAGCAGAGCAGC			
*Ehrlichia*	Primary	EC9	TACCTTGTTACGACTT	*rrs*	477 bp 538 bp	[34]
		EC12A	TGATCCTGGCTCAGAACGAACG			
	Nested	EM87F	GGTTCGCTATTAGTGGCAGA			
		EM584R	CAGTATTAAAAGCCGCTCCA			
	Nested	HF51F	AAGTCGAACGGACAATTACC			
		HF954R	GTTAGGGGGATACGACCTTC			
	Primary	e-gltawf	TTCTCAGGAATACATGCCACC	*gltA*	411 bp	This study
		e-gltawr	ACCATTGAGCAGACCAGCCA			
	Nested	e-gltanf	AATTGCAGGGATAGTGGCAA			
		e-gltanr	CTGTGGCCAAAACCCATCAA			
*Rickettsia*	Primary	R17F1	TTTACAAAATTCTAAAAACCAT	17-kDa protein gene	410 bp	[37]
		RR	TCAATTCACAACTTGCCATT			
	Nested	RrF2	GCTCTTGCAACTTCTATGTT			
		RrR	TCAATTCACAACTTGCCATT			
	Primary	S1	TGATCCTGGCTCAGAACGAAC	*rrs*	1317 bp	[38]
		S2	TAAGGAGGTAATCCAGCCGC			
	Nested	S3	AACACATGCAAGTCGRACGG			
		S4	GGCTGCCTCTTGCGTTAGCT			
	Primary	glta1	TGATCCTGGCTCAGAACGAAC	*gltA*	667 bp	This study
		glta2	TAAGGAGGTAATCCAGCCGC			
	Nested	glta3	AACACATGCAAGTCGRACGG			
		glta4	GGCTGCCTCTTGCGTTAGCT			
		Rr190.70p	ATGGCGAATATTTCTCCAAAA	*ompA*	631 bp	[39]
		Rr190.701n	GTTCCGTTAATGGCAGCATCT			
*R. raoultii*	Primary	Rglta1	ATGACCAATGAAAATAATAAT	*gltA*	341 bp	[40]
		Rglta2	CTTATACTCTCTATGTACA			
	Nested	Rglta3	GGGGACCTGCTCACGGCGG			
		Rglta4	ATTGCAAAAAGTACAGTGAACA			

* Degenerate primer: Y = C or T.

**Table 2:** Prevalence of intracellular tickborne pathogens in ticks collected from hedgehogs in Hubei Province, China.

Tick Species	Year of Tick Collection	Pathogens	Egg Batches (%) n = 20	Dead Engorged Females (%) n = 24	Molted Adults (%) n = 81		Total % n = 125
					MIR	MAR	
*Haemaphysalis flava*	2018	*Anaplasma bovis*	0	20.8	0	0	4
*Haemaphysalis flava*	2018	*Coxiella burnetti*	0	12.5	1.2	8.6	3.2
*Haemaphysalis flava*	2018	*Ehrlichia ewingii*	0	37.5	3.7	25.9	9.6
*Haemaphysalis flava*	2018	*Rickettsia raoultii*	5	0	2.5	17.3	2.4
*Haemaphysalis flava*	2018	*Rickettsia japonica*	0	12.5	1.2	8.6	3.2
		Total	5	83.3	8.6	60.5	22.4

Note: MIR = the minimum infection rate of pooled ticks and MAR=the maximum infection rate of pooled ticks.

## Data Availability

Not applicable.

## References

[1] Eisen L. (2018). Pathogen transmission in relation to duration of attachment by *Ixodes scapularis* ticks. Ticks Tick Borne Dis..

[2] Qiu J.Z., Luo Z.Q. (2017). *Legionella* and *Coxiella* effectors: Strength in diversity and activity. Nat. Rev. Microbiol..

[3] Ben Said M., Belkahia H., Messadi L. (2018). *Anaplasma* spp. in North Africa: A review on molecular epidemiology, associated risk factors and genetic characteristics. Ticks Tick Borne Dis..

[4] Boulanger N., Boyer P., Talagrand-Reboul E., Hansmann Y. (2019). Ticks and tick-borne diseases. Med. Mal. Infect..

[5] Machado-Ferreira E., Vizzoni V.F., Balsemao-Pires E., Moerbeck L., Gazeta G.S., Piesman J., Voloch C.M., Soares C.A.G. (2016). *Coxiella* symbionts are widespread into hard ticks. Parasitol. Res..

[6] El-Mahallawy H.S., Lu G., Kelly P., Xu D., Li Y., Fan W., Wang C. (2015). Q fever in China: A systematic review, 1989–2013. Epidemiol. Infect..

[7] Li J.B., Hu W., Wu T., Li H.B., Hu W.F., Sun Y., Chen Z., Shi Y.L., Zong J., Latif A. (2018). Japanese spotted fever in Eastern China, 2013. Emerg. Infect. Dis..

[8] Jia N., Zheng Y.C., Ma L., Huo Q.B., Ni X.B., Jiang B.G., Chu Y.L., Jiang R.R., Jiang J.F., Cao W.C. (2014). Human infections with *Rickettsia raoultii*, China. Emerg. Infect. Dis..

[9] Jung M., Kho J.W., Lee W.G., Roh J.Y., Lee D.H. (2019). Seasonal occurrence of *Haemaphysalis longicornis* (Acari: Ixodidae) and *Haemaphysalis flava*, vectors of severe fever with thrombocytopenia syndrome (SFTS) in South Korea. J. Med. Entomol..

[10] Li Z.B., Cheng T.Y., Xu X.L., Song L.L., Liu G.H. (2017). Genetic variation in mitochondrial genes of the tick *Haemaphysalis flava* collected from wild hedgehogs in China. Exp. Appl. Acarol..

[11] Chae J.B., Kang J.G., Kim H.C., Chong S.T., Lee I.Y., Shin N.S., Chae J.S. (2017). Identification of tick species collected from wild boars and habitats of wild boars and domestic pigs in the Republic of Korea. Korean J. Parasitol..

[12] Duan D., Cheng T. (2017). Determination of the microbial community features of *Haemaphysalis flava* in different developmental stages by high-throughput sequencing. J. Basic Microbiol..

[13] Kim H.C., Han S.H., Chong S.T., Klein T.A., Choi C.Y., Nam H.Y., Chae H.Y., Lee H., Ko S., Kang J.G. (2011). Ticks collected from selected mammalian hosts surveyed in the Republic of Korea during 2008–2009. Korean J. Parasitol..

[14] Cheng W.Y., Zhao G.H., Jia Y.Q., Bian Q.Q., Du S.Z., Fang Y.Q., Qi M.Z., Yu S.K. (2013). Characterization of *Haemaphysalis flava* (Acari: Ixodidae) from Qingling subspecies of Giant Panda (*Ailuropoda melanoleuca qinlingensis*) in Qinling Mountains (Central China) by morphology and molecular markers. PLoS ONE.

[15] Deng G.F. (1991). Economic Insect Fauna of China.

[16] Shi X.Q., Zhou Z.Y. (1991). The discovery of the *Haemaphysalis flava* on hedgehog surface in Shanghai. Shanghai J. Anim. Husb. Vet. Med..

[17] Ejiri H., Lim C.K., Isawa H., Yamaguchi Y., Fujita R., Takayama-Ito M., Kuwata R., Kobayashi D., Horiya M., Posadas-Herrera G. (2018). Isolation and characterization of Kabuto Mountain virus, a new tick-borne phlebovirus from *Haemaphysalis flava* ticks in Japan. Virus Res..

[18] Fujita R., Ejiri H., Lim C.K., Noda S., Yamauchi T., Watanabe M., Kobayashi D., Takayama-Ito M., Murota K., Posadas-Herrera G. (2017). Isolation and characterization of Tarumizu tick virus: A new coltivirus from *Haemaphysalis flava* ticks in Japan. Virus Res..

[19] Hong S.H., Kim S.Y., Song B.G., Rho J.R., Cho C.R., Kim C.N., Um T.H., Kwak Y.G., Cho S.H., Lee S.E. (2019). Detection and characterization of an emerging type of *Babesia sp.* similar to *Babesia motasi* for the first case of human babesiosis and ticks in Korea. Emerg. Microbes Infect..

[20] Jo Y.S., Kang J.G., Chae J.B., Cho Y.K., Shin J.H., Jheong W.H., Chae J.S. (2019). Prevalence of severe fever with thrombocytopenia syndrome virus in ticks collected from National Parks in Korea. Vector Borne Zoonotic Dis..

[21] Kang J.G., Ko S., Kim H.C., Chong S.T., Klein T.A., Chae J.B., Jo Y.S., Choi K.S., Yu D.H., Park B.K. (2016). Prevalence of anaplasma and *Bartonella* spp. in ticks collected from Korean water deer (*Hydropotes inermis argyropus*). Korean J. Parasitol..

[22] Suzuki J., Hashino M., Matsumoto S., Takano A., Kawabata H., Takada N., Andoh M., Oikawa Y., Kajita H., Uda A. (2016). Detection of *Francisella tularensis* and analysis of bacterial growth in ticks in Japan. Lett. Appl. Microbiol..

[23] Takhampunya R., Kim H.C., Chong S.T., Korkusol A., Tippayachai B., Davidson S.A., Petersen J.M., Klein T.A. (2017). *Francisella*-like endosymbiont detected in *Haemaphysalis* ticks (Acari: Ixodidae) from the Republic of Korea. J. Med. Entomol..

[24] Yun S.M., Lee Y.J., Choi W., Kim H.C., Chong S.T., Chang K.S., Coburn J.M., Klein T.A., Lee W.J. (2016). Molecular detection of severe fever with thrombocytopenia syndrome and tick-borne encephalitis viruses in ixodid ticks collected from vegetation, Republic of Korea, 2014. Ticks Tick Borne Dis..

[25] Kim J.Y., Kwak Y.S., Lee I.Y., Yong T.S. (2020). Molecular detection of *Toxoplasma Gondii* in *Haemaphysalis* ticks in Korea. Korean J. Parasitol..

[26] Lu M., Tian J.H., Yu B., Guo W.P., Holmes E.C., Zhang Y.Z. (2017). Extensive diversity of rickettsiales bacteria in ticks from Wuhan, China. Ticks Tick Borne Dis..

[27] Zheng W.Q., Xuan X.N., Fu R.L., Tao H.Y., Liu Y.Q., Liu X.Q., Li D.M., Ma H.M., Chen H.Y. (2018). Tick-borne pathogens in Ixodid ticks from Poyang lake region, Southeastern China. Korean J. Parasitol..

[28] Levy S. (2013). The Lyme disease debate: Host biodiversity and human disease risk. Environ. Health Perspect..

[29] Gong X.Q., Xiao X., Liu J.W., Han H.J., Qin X.R., Lei S.C., Yu X.J. (2020). Occurrence and genotyping of *Coxiella burnetii* in hedgehogs in China. Vector Borne Zoonotic Dis..

[30] Luo L.M., Zhao L., Wen H.L., Zhang Z.T., Liu J.W., Fang L.Z., Xue Z.F., Ma D.Q., Zhang X.S., Ding S.J. (2015). *Haemaphysalis longicornis* ticks as reservoir and vector of severe fever with thrombocytopenia syndrome virus in China. Emerg. Infect. Dis..

[31] Li L.H., Zhu D., Zhang C.C., Zhang Y., Zhou X.N. (2016). Experimental transmission of *Babesia microti* by *Rhipicephalus haemaphysaloides*. Parasites Vectors.

[32] Han H.J., Liu J.W., Wen H.L., Qin X.R., Zhao M., Wang L.J., Zhou C.M., Qi R., Yu H., Yu X.J. (2018). *Babesia vesperuginis* in insectivorous bats from China. Parasites Vectors.

[33] Chitanga S., Simulundu E., Simuunza M.C., Changula K., Qiu Y., Kajihara M., Nakao R., Syakalima M., Takada A., Mweene A.S. (2018). First molecular detection and genetic characterization of *Coxiella burnetii* in Zambian dogs and rodents. Parasites Vectors.

[34] Kawahara M., Rikihisa Y., Lin Q., Isogai E., Tahara K., Itagaki A., Hiramitsu Y., Tajima T. (2006). Novel genetic variants of *Anaplasma phagocytophilum*, *Anaplasma bovis*, *Anaplasma centrale*, and a novel *Ehrlichia sp.* in wild deer and ticks on two major islands in Japan. Appl. Environ. Microbiol..

[35] Weisburg W.G., Barns S.M., Pelletier D.A., Lane D.J. (1991). 16S ribosomal DNA amplification for phylogenetic study. J. Bacteriol..

[36] Parola P., Roux V., Camicas J.L., Baradji I., Brouqui P., Raoult D. (2000). Detection of ehrlichiae in African ticks by polymerase chain reaction. Trans. R. Soc. Trop. Med. Hyg..

[37] Zhang X., Geng J., Du J., Wang Y., Qian W., Zheng A., Zou Z. (2018). Molecular identification of Rickettsia species in *Haemaphysalis* ticks collected from Southwest China. Vector Borne Zoonotic Dis..

[38] Huang Y., Zhao L., Zhang Z., Liu M., Xue Z., Ma D., Sun X., Sun Y., Zhou C., Qin X. (2017). Detection of a novel *Rickettsia* from *Leptotrombidium scutellare* mites (Acari: Trombiculidae) from Shandong of China. J. Med. Entomol..

[39] Roux V., Fournier P.E., Raoult D. (1996). Differentiation of spotted fever group rickettsiae by sequencing and analysis of restriction fragment length polymorphism of PCR-amplified DNA of the gene encoding the protein rOmpA. J. Clin. Microbiol..

[40] Zhuang L., Du J., Cui X.M., Li H., Tang F., Zhang P.H., Hu J.G., Tong Y.G., Feng Z.C., Liu W. (2018). Identification of tick-borne pathogen diversity by metagenomic analysis in *Haemaphysalis longicornis* from Xinyang, China. Infect. Dis. Poverty.

[41] Luo L.M., Sun J.M., Yan J.B., Wang C.W., Zhang Z.T., Zhao L., Han H.J., Tong Z.D., Liu M.M., Wu Y.Y. (2016). Detection of a novel *Ehrlichia* Species in *Haemaphysalis longicornis* tick from China. Vector Borne Zoonotic Dis..

[42] Singh K.R., Pavri K., Anderson C.R. (1963). Experimental transovarial transmission of Kyasanur forest disease virus in *Haemaphysalis spinigera*. Nature.

[43] Burgdorfer W., Brinton L.P. (1975). Mechanisms of transovarial infection of spotted fever Rickettsiae in ticks. Ann. N. Y. Acad. Sci..

[44] Starkey L.A., Barrett A.W., Beall M.J., Chandrashekar R., Thatcher B., Tyrrell P., Little S.E. (2015). Persistent *Ehrlichia ewingii* infection in dogs after natural tick infestation. J. Vet. Intern. Med..

[45] Stuen S., Nevland S., Moum T. (2003). Fatal cases of tick-borne fever (TBF) in sheep caused by several 16S rRNA gene variants of *Anaplasma phagocytophilum*. Ann. N. Y. Acad. Sci..

[46] Tay S.T., Koh F.X., Kho K.L., Sitam F.T. (2015). Rickettsial infections in monkeys, Malaysia. Emerg. Infect. Dis..

[47] Sun J.M., Lin J.F., Gong Z.Y., Chang Y., Ye X.D., Gu S.P., Pang W.L., Wang C.W., Zheng X.H., Hou J. (2015). Detection of spotted fever group *Rickettsiae* in ticks from Zhejiang Province, China. Exp. Appl. Acarol..

[48] Qin X.R., Han H.J., Han F.J., Zhao F.M., Zhang Z.T., Xue Z.F., Ma D.Q., Qi R., Zhao M., Wang L.J. (2019). Rickettsia japonica and novel *Rickettsia* species in ticks, China. Emerg. Infect. Dis..

[49] Wen J., Jiao D., Wang J.H., Yao D.H., Liu Z.X., Zhao G., Ju W.D., Cheng C., Li Y.J., Sun Y. (2014). *Rickettsia raoultii*, the predominant *Rickettsia* found in *Dermacentor silvarum* ticks in China–Russia border areas. Exp. Appl. Acarol..

[50] Guo L.P., Mu L.M., Xu J., Jiang S.H., Wang A.D., Chen C.F., Guo G., Zhang W.J., Wang Y.Z. (2015). *Rickettsia raoultii* in *Haemaphysalis erinacei* from marbled polecats, China-Kazakhstan border. Parasites Vectors.

[51] Dong Z.H., Yang Y.C., Wang Q., Xie S.S., Zhao S.S., Tan W.B., Yuan W.M., Wang Y.Z. (2019). A case with neurological abnormalities caused by *Rickettsia raoultii* in northwestern China. BMC Infect. Dis..

[52] Liu H., Liang X.T., Wang H.J., Sun X.T., Bai X., Hu B., Shi N., Wang N., Zhang X.L., Huang L.Z. (2020). Molecular evidence of the spotted fever group *Rickettsiae* in ticks from Yunnan Province, Southwest China. Exp. Appl. Acarol..

[53] Gaowa W., Yin X.H., Guo S.C., Ding C.L., Cao M.Z., Kawabata H., Sato K., Ando S., Fujita H., Kawamori F. (2018). Spotted fever group *Rickettsiae* in Inner Mongolia, China, 2015–2016. Emerg. Infect. Dis..

[54] Gaywee J., Sunyakumthorn P., Rodkvamtook W., Ruang-Areerate T., Mason C.J., Sirisopana N. (2007). Human infection with *Rickettsia sp.* related to *R. japonica*, Thailand. Emerg. Infect. Dis..

[55] Jiang J., Choi Y.J., Kim J., Kim H.C., Klein T.A., Chong S.T., Richards A.L., Park H.J., Shin S.H., Song D. (2019). Distribution of *Rickettsia* spp. in ticks from Northwestern and Southwestern Provinces, Republic of Korea. Korean J. Parasitol..

[56] Seo M.G., Kwon O.D., Kwak D. (2020). High prevalence of *Rickettsia raoultii* and associated pathogens in canine ticks, South Korea. Emerg. Infect. Dis..

[57] Ishikura M., Ando S., Shinagawa Y., Matsuura K., Hasegawa S., Nakayama T., Fujita H., Watanabe M. (2003). Phylogenetic analysis of spotted fever group rickettsiae based on gltA, 17-kDa, and rOmpA genes amplified by nested PCR from ticks in Japan. Microbiol. Immunol..

[58] Li W., Liu L., Jiang X., Guo X., Garnier M., Raoult D., Parola P. (2009). Molecular identification of spotted fever group Rickettsiae in ticks collected in central China. Clin. Microbiol. Infect..

[59] Fournier P.E., Fujita H., Takada N., Raoult D. (2002). Genetic identification of rickettsiae isolated from ticks in Japan. J. Clin. Microbiol..

